# HIV Testing, Social Capital, and Mental Health Access Among Foreign-Born Men Who Have Sex with Men (MSM) in Japan

**DOI:** 10.3390/healthcare14040520

**Published:** 2026-02-18

**Authors:** Adam O. Hill, Thomas Norman, Amal R. Khanolkar, Kohta Iwahashi, Noriyo Kaneko

**Affiliations:** 1 School of Medicine, Nagoya City University, Nagoya 467-8601, Japan; noriyok@med.nagoya-cu.ac.jp; 2 Australian Research Centre in Sex, Health and Society, La Trobe University, Bundoora, VIC 3086, Australia; t.norman@latrobe.edu.au; 3 Department of Population Health Sciences, School of Life Course and Population Sciences, King’s College London, London SE1 1UL, UK; amal.khanolkar@kcl.ac.uk; 4 NPO AKTA, Tokyo 160-0022, Japan; iwahashi@akta.jp

**Keywords:** MSM, social capital, HIV testing, mental health access, country of birth, Japan, health disparities

## Abstract

**Highlights:**

Country of birth was associated with distinct patterns of social capital, disclosure, and health-related access, underscoring the importance of structurally informed and evidence-based prevention strategies. Foreign-born MSM in Japan demonstrated higher engagement with HIV testing despite more limited access to community infrastructure and mental health services.

**Abstract:**

Background: Migration and place of birth are increasingly recognised as social determinants of health among sexual minority populations. Among men who have sex with men (MSM), being born outside the country of residence may shape access to healthcare, community resources, and social capital networks. In Japan, however, little is known about how being born outside Japan is associated with social capital, health behaviours, and mental health among MSM. Methods: Data were drawn from a large cross-sectional online survey conducted in 2025 of 8150 MSM living in Japan, recruited via community-based in-person outreach and targeted geo-social networking application advertisements. Multivariable logistic regression analyses examined associations between country of birth and social, behavioural, and health-related outcomes. Results: Foreign-born MSM were younger and more concentrated in the Greater Tokyo metropolitan region. Being born outside Japan was associated with higher odds of HIV testing across all timeframes and higher levels of both gay and heterosexual social capital. Foreign-born MSM were also more likely to have disclosed their sexuality to friends and family. However, they were less likely to be aware of LGBT or HIV prevention organisations, despite higher participation once engaged. No differences were observed in suicidal ideation or unprotected anal intercourse with casual partners, although foreign-born MSM were more likely to report unmet need for mental health care. Conclusions: Foreign-born MSM in Japan demonstrate strong engagement in HIV prevention and higher social capital, alongside persistent barriers to community awareness and mental health service access. These findings highlight the importance of addressing structural and informational barriers and supporting community-based organisations to improve equitable health and wellbeing outcomes among MSM in Japan.

## 1. Introduction

Migration and place of birth are increasingly recognised as important social determinants of health, shaping access to healthcare, social integration, and exposure to structural vulnerability [1,2]. Among men who have sex with men (MSM), migration-related positioning has been associated with differences in HIV testing, disclosure, community engagement, quality of life, and access to prevention services across a range of international contexts [3,4,5,6]. Notably, such differences are not consistently explained by variations in individual risk-taking behaviours. Rather, a growing body of research points to the role of social positioning, access to resources, and unequal distributions of social capital in shaping health-related opportunities and constraints among sexual minority men [7,8,9].

Bourdieusian social capital theory provides a useful framework for motivating the inclusion and measurement of social capital in this study. Social capital is conceptualised as access to valued resources embedded within social relationships that individuals may draw upon through their different networks, in this case, operationalised separately as ‘gay’ and ‘heterosexual’ social networks. [10,11]. Among MSM, previous research has demonstrated that social capital operates differently depending on whether resources are accessed through gay or heterosexual social networks, with these forms of social capital showing distinct associations with suicidal ideation, self-rated health and happiness, and HIV-risk-related behaviours [8,9,12,13].

Japan provides a distinctive context in which to examine migration status and social capital among MSM. Despite universal health coverage, the HIV epidemic remains concentrated among MSM, with one-third (33.4%) of HIV diagnoses presenting as AIDS in 2024 [14], and ongoing challenges in prevention and testing access [15,16,17]. Over the past two decades, Japan has experienced a steady increase in its non-Japanese population, particularly among working-age adults, reaching 3.1% of the total population [18]. National HIV surveillance data, which are reported by nationality rather than place of birth, indicate that non-Japanese nationals account for a disproportionate share of new HIV and AIDS diagnoses. In 2024, foreign nationals accounted for nearly one-fifth (19.3%) of the total new infections in Japan, with 16.8% of newly diagnosed HIV infections and 18.9% of male AIDS diagnoses, despite comprising a relatively small proportion of the population [18]. While these population-level statistics are reported by nationality, this study uses country of birth as an indicator of migration-related social positioning, recognising that nationality alone may not capture the social and networked dimensions of migration relevant to health. Country of birth offers a more stable and analytically tractable marker of early socialisation and migration experience, which may shape language use, social networks, and access to community resources in ways that persist beyond formal citizenship status. Nevertheless, migration status has rarely been examined analytically in Japanese MSM research, and when it is included, it is often treated as a residual demographic characteristic rather than a structural position shaping access to social and community resources. This has limited understanding of how being foreign-born may influence the accumulation and mobilisation of different forms of social capital among MSM in Japan.

Community-based and peer-led organisations play a central role in the production and circulation of gay social capital, including access to HIV prevention information, testing opportunities, peer norms, and psychosocial support [8,19]. At the same time, heterosexual social capital, embedded in family, workplace, and broader social networks, may confer different advantages and constraints, particularly in relation to disclosure and social integration [9,20]. In Japan, LGBT and HIV-focused community organisations operate within a context of limited funding and increasing precarity [21], raising concerns about whether these resources are equitably accessible to MSM who are less embedded in local gay communities, including those who are foreign-born or geographically mobile. It is important to note that ‘foreign born’ in this study does not equate to non-Japanese nationality or limited language proficiency. Individuals born outside Japan may hold Japanese citizenship, permanent residency, or other legal statuses, and may have spent much or most of their lives in Japan. It is important to note that ‘foreign born’ in this study does not equate to non-Japanese nationality or limited language proficiency. Individuals born outside Japan may hold Japanese citizenship, permanent residency, or other legal statuses, and may have spent much or most of their lives in Japan. The survey was administered in Japanese, indicating functional Japanese literacy among participants, although some respondents may have used translation tools or assistance. Country of birth is therefore conceptualised here as an analytic indicator of migration-related social positioning, rather than as a proxy for nationality, legal status, or language ability, consistent with research framing migration as a structural social determinant of health [1,2,4].

Despite growing international attention to migration and sexual minority health, little is known about how being born outside Japan is associated with social positioning, access to social capital, and health-related outcomes among MSM at a national level. Using data from the largest national survey of MSM conducted in Japan to date (n = 8150), this study examines whether being born outside Japan is associated with a range of social, behavioural, and health-related outcomes. Specifically, we examine associations between country of birth and disclosure, awareness of and participation in LGBT and HIV prevention organisations, access to gay and heterosexual social capital, HIV testing (past 6 months, past 12 months, and lifetime), unprotected anal intercourse with casual partners, suicidal ideation, and unmet need for mental health care. Country of birth is treated as the focal explanatory variable across analyses, with sociodemographic characteristics included as covariates. Each outcome is analysed separately, with outcomes grouped by domain for presentation.

## 2. Materials and Methods

### Data Sample and Procedure

Data for this study were drawn from a cross-sectional, anonymous online survey examining health, behavioural practices, and community engagement among men who have sex with men (MSM) living in Japan. The survey was conducted between 31 May and 14 June 2025. Eligibility criteria required participants to be aged 18 years or older, currently residing in Japan, and to identify as gay, bisexual, or otherwise same sex attracted, or to report same sex experience. Prior to commencing the survey, participants were presented with a detailed study description and were required to confirm eligibility and provide informed consent by selecting two mandatory consent items. Participants were offered the opportunity to enter a prize draw for vouchers valued at 1000 to 2000 yen (approximately 7–14 USD). No names, contact details, or other direct personal identifiers were collected as part of the survey. Survey responses were collected via a secure online platform and were accessible only to members of the research team. Participants were able to skip any non-mandatory items and could exit the survey at any time prior to submission. For the optional prize draw, participants were redirected to a separate form that was not linked to survey responses, ensuring the anonymity of survey data.

Participants were recruited using two parallel strategies. First, in-person recruitment was conducted at a large gay community-based event held in central Nagoya, Aichi Prefecture, between 31 May and 1 June 2025. Event attendees were provided with brief study information and invited to complete the survey on site using their personal smartphones. Second, targeted paid advertisements were placed on 9Monsters, the most widely used geo-social networking application among MSM in Japan [22]. Two versions of the advertisement were deployed as full-screen splash advertisements displayed upon application opening. One featured a shirtless male model, while the other used a community-oriented design incorporating a rainbow flag. Both advertisements used identical text and design language and were produced by the same designer. Each advertisement linked directly to the online survey.

In total, the survey link was accessed 38,500 times, resulting in 8150 completed surveys. The male model-themed advertisement was viewed 829,731 times and generated 13,568 clicks, yielding 3089 completed surveys. The community-oriented advertisement was viewed 823,353 times and generated 15,022 clicks, yielding 4544 completed surveys. Following data cleaning procedures that excluded incomplete responses, participants not residing in Japan, and respondents not assigned male at birth, the analytic dataset comprised 8077 valid participants. For the purposes of this manuscript, participants who did not report annual income were excluded. The final analytic sample consisted of 8025 participants for descriptive analyses and 7662 participants for the multivariable logistic regression examining sociodemographic correlates of being foreign-born among MSM in Japan (Model 1). Ethical approval for the study was obtained from the Nagoya City University Human Research Ethics Committee (approval number 24008-2).

## 3. Materials

The survey instrument was developed specifically for this study and informed by existing behavioural surveillance instruments used in Japan and in international MSM HIV research [8,23,24,25]. Questionnaire development involved collaboration between Japanese and international HIV prevention researchers and leaders and advisors from gay community NPOs to ensure cultural, linguistic, and contextual appropriateness. The survey was administered exclusively in Japanese. Interpretation and translation of findings into English were conducted by bilingual members of the research team, including senior authors.

Outcome variables were selected a priori based on prior literature on MSM health, migration, social capital, and HIV prevention, and grouped by domain for clarity of presentation. The following variables from the survey were used in the present analyses.

### 3.1. Demographic Variables and Recruitment Method (Model 1)

Age was collected as a continuous variable. Age was categorised following conventions used in previous Japanese MSM research [8,13]. Age was retained as a continuous variable in all multivariable regression models.

Sexual orientation was assessed by asking participants to select the term that best described their sexuality from a list including ‘gay’, ‘lesbian’, ‘bisexual’, ‘heterosexual’, ‘don’t know’, ‘prefer not to decide’, and ‘something else’. Participants not assigned male at birth and those identifying as heterosexual were excluded during data cleaning, as they did not meet study eligibility criteria. For analysis, responses of ‘don’t know’, ‘prefer not to decide’, and ‘something else’ were combined into a single ‘something else’ category due to conceptual overlap and small cell sizes. Final categories used in analysis were ‘gay’, ‘bisexual’, and ‘something else’.

Country of birth was assessed by asking participants whether they were born in Japan or outside Japan. Participants who reported being born outside Japan were invited to specify their country of birth using a free-text response. For analyses, country of birth was used as a binary variable indicating birth in Japan versus being born outside Japan.

Employment status was measured using multiple response options. For analysis, responses were grouped into five categories: full-time employee, part-time or casual employee, student, unemployed, and other.

Annual income was assessed using income brackets aligned with Japanese taxation and labour reporting standards. Response options included: no income; less than 2 million yen (>15,000 USD); 2 to below 4 million yen (>30,000 USD); 4 to below 6 million yen (30,000 USD to >45,000 USD); 6 to below 8 million yen (45,000 USD–>60,000 USD); 8 to below 10 million yen (60,000 USD–>75,000 USD); 10 million yen or more (75,000 USD+); and do not know or prefer not to say. For analysis, income was recoded into four categories: no income; below 2 million yen; 2 to below 6 million yen; and 6 million yen or more. Participants selecting ‘don’t know or prefer not to say’ were excluded from analyses involving income (n = 342).

Residential location was assessed by asking participants to report their current prefecture of residence. Prefectures were grouped into eight standard geographic regions of Japan: Kanto, Kansai, Chubu, Kyushu and Okinawa, Tohoku, Chugoku, Hokkaido, and Shikoku. This regional classification follows established administrative divisions and has been used in previous research examining HIV testing behaviours and access to community resources in Japan [25,26].

Recruitment method was categorised as in-person recruitment, geo-social networking application advertisement featuring a male model, or geo-social networking application community-oriented advertisement, as described in Data Sample and Procedure Section.

### 3.2. Disclosure of Sexual Orientation

Disclosure of sexual orientation to friends was assessed by asking participants how many of their friends were aware of their sexuality. Response options included: no one; one to two people; three to four people; five or more people; and open to all. Disclosure of sexual orientation to family members was assessed using an identical response structure. For analytic purposes, both disclosure variables were dichotomised to indicate disclosure to at least one person versus disclosure to no one.

### 3.3. Community Organisation Awareness and Participation

Participants were asked whether they were aware of any community centres or organisations involved in LGBT-related HIV prevention activities in Japan. A list of examples of community and HIV prevention organisations operating in Japan was provided to help participant recall. Responses were recorded as ‘yes’ or ‘no’. Those who responded ‘yes’ were then asked whether they had participated in any programmes either across their lifetime or in the past 12 months.

### 3.4. HIV Risk-Taking and Preventative Behaviours

HIV testing was assessed by asking participants to self-report if they had been tested for HIV in the past 6 months, in the past 12 months, and at any point in their lifetime. Using three binary outcome variables indicating whether participants had been tested for HIV in the past 6 months, in the past 12 months, and at any point in their lifetime.

UAI with casual partners in the past 6 months was derived from a composite measure based on responses to multiple survey items. Participants were classified as having engaged in UAI if they reported anal intercourse with a casual partner in the past 6 months and met all of the following criteria: they did not always use condoms during anal sex, they were not using pre-exposure prophylaxis (PrEP) at the time and neither was their sexual partner, and they were not living with HIV with a confirmed undetectable viral load.

### 3.5. Mental Health

Recent suicidal ideation was assessed using item 9 of the Patient Health Questionnaire-9 (PHQ-9) [27,28], which has been validated for use in Japan [29]. Participants were asked how often, during the past two weeks, they had been bothered by thoughts that they would be better off dead or of harming themselves in some way. Response options included not at all, several days, more than half the days, and nearly every day. For analysis, responses of several days, more than half the days, and nearly every day were combined to indicate the presence of suicidal ideation and compared with those who responded not at all.

Mental health service contact was assessed by asking participants whether they had ever received psychiatric or psychosomatic treatment, or whether they had ever wanted to receive such treatment but had been unable to do so or had discontinued attempts to access care.

### 3.6. Social Capital

Individual social capital was measured using the Resource Generator [30,31], a validated instrument for assessing valued resources through social networks [32]. Social capital scores were derived from 18 items, with one point assigned per item, yielding a possible range from 0 to 18. Example items included ‘Do you have access to someone who could do the shopping for you if you were ill and unable to?’ and ‘Do you have access to someone whom you trust?’ Participants indicated whether each resource was accessed through MSM, heterosexual individuals, both MSM and heterosexual individuals, or neither. For analysis, participants were categorised as having high social capital if their score was at least one standard deviation above the sample mean, low social capital if their score was at least one standard deviation below the mean, and medium social capital if their score fell between these thresholds as in previous research [8,13].

### 3.7. Statistical Analyses

All statistical analyses were conducted using Stata version 18 SE (StataCorp, College Station, TX, USA). Firstly, logistic regression analyses were used to examine associations between participant characteristics and country of birth (binary outcome distinguishing participants born in Japan from those born outside Japan). Univariable logistic regression models were used to assess unadjusted associations between each independent variable (age, sexual orientation, recruitment modality, employment status, income, and region of residence) and country of birth. Unadjusted odds ratios (ORs), 95% confidence intervals (CIs), and *p*-values are reported. Multivariable logistic regression models were then used to examine mutually adjusted associations between independent variables and country of birth.

Secondly, multivariable logistic regression models were also used to examine associations between country of birth and all outcomes of interest. The first model examined associations between country of birth and health-related, behavioural, disclosure, social capital, and community engagement variables, including HIV testing history (past 6 months, past 12 months, and lifetime), engagement in UAI with casual partners in the past 6 months, suicidal ideation in the past two weeks, history of psychiatric or psychosomatic treatment or unmet need for such treatment. A second multivariable logistic regression model examined associations between country of birth and disclosure of sexual orientation to friends and family, awareness of and participation in LGBT or HIV prevention organisations in the past 12 months, and levels of gay and heterosexual social capital. Both logistic models were adjusted for sociodemographic variables included in Model 1. Each outcome was analysed in a separate multivariable logistic regression adjusted for sociodemographic characteristics, and outcomes were not adjusted for one another.

Variables with a *p*-value below 0.25 in univariable analyses were considered for inclusion in multivariable models. This threshold was used to ensure that variables potentially associated with country of birth were not excluded prematurely and is consistent with established modelling practices [33]. Multicollinearity among independent variables was assessed using variance inflation factors (VIFs). All VIF values were below 2, indicating no evidence of problematic multicollinearity. Adjusted odds ratios (AORs), 95% confidence intervals, and *p*-values were reported for all multivariable regression models.

## 4. Results

### 4.1. Descriptive Statistics (Table 1) Presents Baseline Sociodemographic Characteristics, Social Positioning Variables, and Health-Related Outcomes by Country of Birth

Table 1 presents descriptive characteristics of the study sample by country of birth. The sample comprised 8025 MSM, of whom 245 (3.1%) were born outside Japan. Among foreign-born participants, the most common countries of birth were China (25.7%, n = 63), Taiwan (9.4%, n = 23), the United States (7.8%, n = 19), Brazil (7.4%, n = 18), the Republic of Korea (6.5%, n = 16), and the Philippines (6.1%, n = 15).

Foreign-born participants tended to be younger than those born in Japan, with a higher proportion aged 25–34 years (43.3% vs. 20.7%) and a lower proportion aged 45 years or older (11.0% vs. 43.7%). A higher proportion of foreign-born participants identified as gay compared with Japan-born participants (81.2% vs. 75.8%).

Employment profiles differed, with foreign-born participants more likely to be employed full-time (71.0% vs. 64.2%) or unemployed (13.1% vs. 4.8%), and less likely to be in part-time or casual employment (3.3% vs. 7.8%). Geographic distribution also varied by country of birth. Foreign-born participants were more concentrated in Kanto (Greater Tokyo) (62.0% vs. 43.7%) and less likely to reside in non-metropolitan regions.

Patterns of sexuality disclosure differed markedly. Foreign-born participants were more likely to have disclosed their sexuality to friends (85.3% vs. 70.7%) and to family members (46.5% vs. 29.3%). However, they were less likely to report awareness of LGBT or AIDS prevention organisations (35.5% vs. 56.0%), despite being more likely to report participation in prevention programmes in the past 12 months (9.8% vs. 4.8%).

Differences were also observed for social capital and health-related outcomes. Foreign-born participants were more likely to report high levels of gay social capital (33.9% vs. 23.2%) and high levels of heterosexual social capital (37.6% vs. 23.1%). They were also more likely to report recent HIV testing, including testing in the past 6 months (39.6% vs. 24.9%), the past 12 months (52.2% vs. 33.9%), and lifetime testing (77.1% vs. 68.1%). Lastly, foreign-born participants were less likely to report having received psychiatric or psychosomatic treatment (29.2% vs. 35.9%), but more likely to report having wanted such treatment but having given up accessing it (24.3% vs. 16.7%).

**Table 1:** Descriptive statistics for all study variables.

	Born in Japan		Foreign Born		All Participants	
Variable	(n)	(%)	(n)	(%)	(n)	(%)
**Age group (years)**						
18–24 years	676	8.7	30	12.2	706	8.8
25–34 years	1610	20.7	106	43.3	1716	21.4
35–44 years	2087	26.9	82	33.5	2169	27.1
45–54 years	2262	29.1	18	7.3	2280	28.4
55 years+	1136	14.6	9	3.7	1145	14.3
**Sexuality**						
Gay	5895	75.8	199	81.2	6094	75.9
Bisexual	1544	19.8	39	15.9	1583	19.7
Something else	341	4.4	7	2.9	348	4.3
**Country of birth**						
Japan	7780	100.0	0	100.0	7780	96.9
Other	0	0.0	245	0.0	245	3.1
**Recruitment group**						
In-person recruitment	3003	38.6	71	29.0	3074	38.3
Geo-social networking app advertisement (male model)	4346	55.9	166	67.8	4512	56.2
Geo-social networking app community advertisement	431	5.5	8	3.3	439	5.5
**Employment**						
Full-time employee	4993	64.2	174	71.0	5167	64.4
Part-time/casual employee	603	7.8	8	3.3	611	7.6
Student	489	6.3	10	4.1	499	6.2
Unemployed	375	4.8	32	13.1	407	5.1
Other	1320	17.0	21	8.6	1341	16.7
**Income**						
<2 million yen	240	3.2	9	3.9	249	3.2
No income	974	13.1	36	15.6	1010	13.1
2–5.99 million yen	4322	57.9	126	54.5	4448	57.8
6 million yen+	1923	25.8	60	26.0	1983	25.8
**Residential location**						
Kanto	3390	43.7	150	62.0	3540	44.3
Kansai	1321	17.0	39	16.1	1360	17.0
Chubu	1236	15.9	35	14.5	1271	15.9
Kyushu/Okinawa	760	9.8	7	2.9	767	9.6
Tohoku	353	4.6	2	0.8	355	4.4
Chugoku	278	3.6	5	2.1	283	3.5
Hokkaido	273	3.5	3	1.2	276	3.5
Shikoku	144	1.9	1	0.4	145	1.8
**Disclosed sexuality to friends**						
No	2277	29.3	36	14.7	2313	28.8
Yes	5499	70.7	209	85.3	5708	71.2
**Disclosed sexuality to family**						
No	5491	70.7	131	53.5	5622	70.1
Yes	2281	29.3	114	46.5	2395	29.9
**Aware of LGBT/AIDS prevention community organisation**						
No	3426	44.0	158	64.5	3584	44.7
yes	4354	56.0	87	35.5	4441	55.3
**Participated in LGBT/AIDS prevention org programme (past 12 months)**						
No	7404	95.2	221	90.2	7625	95.0
yes	376	4.8	24	9.8	400	5.0
**Gay social capital**						
Low gay social capital	2474	31.8	66	26.9	2540	31.7
Medium gay social capital	3499	45.0	96	39.2	3595	44.8
High gay social capital	1807	23.2	83	33.9	1890	23.6
**Heterosexual social capital**						
Low heterosexual social capital	2129	27.4	40	16.3	2169	27.0
Medium heterosexual social capital	3856	49.6	113	46.1	3969	49.5
High heterosexual social capital	1795	23.1	92	37.6	1887	23.5
**HIV test in past 6 months**						
No	5845	75.1	148	60.4	5993	74.7
Yes	1935	24.9	97	39.6	2032	25.3
**HIV test in past 12 months**						
No	5142	66.1	117	47.8	5259	65.5
Yes	2638	33.9	128	52.2	2766	34.5
**HIV test ever in lifetime**						
No	2485	31.9	56	22.9	2541	31.7
Yes	5295	68.1	189	77.1	5484	68.3
**UAI with casual partner in past 6 months**						
No	2073	47.3	90	51.4	2163	47.4
Yes	2311	52.7	85	48.6	2396	52.6
**Suicidal ideation in past two weeks**						
No	5389	69.3	167	68.2	5556	69.3
Yes	2388	30.7	78	31.8	2466	30.7
**Ever received treatment from a psychiatrist or psychosomatic medicine specialist**						
No	4972	64.1	172	70.8	5144	64.3
Yes	2785	35.9	71	29.2	2856	35.7
**Ever wanted psychiatric or psychosomatic treatment but gave up**						
No	6463	83.3	184	75.7	6647	83.1
Yes	1295	16.7	59	24.3	1354	16.9

### 4.2. Sociodemographic Correlates of Being Foreign-Born (Table 2)

This study examines multiple outcome domains rather than a single primary outcome, reflecting its exploratory aims. In multivariable analyses (Table 2), younger age was associated with being foreign-born (AOR per year increase = 0.94, 95% CI: 0.93–0.95, *p* < 0.001). Compared with gay men, bisexual men had lower odds of being foreign-born (AOR = 0.58, 95% CI: 0.40–0.85, *p* = 0.005), as did those identifying with another sexuality (AOR = 0.36, 95% CI: 0.14–0.91, *p* = 0.032).

Participants recruited via a community-oriented geo-social networking application advertisement had higher odds of being foreign-born compared with in-person recruitment (AOR = 2.73, 95% CI: 1.20–6.18, *p* = 0.016).

Part-time/casual employment (AOR = 0.38, 95% CI: 0.16–0.88, *p* = 0.025) and ‘other’ employment (AOR = 0.52, 95% CI: 0.31–0.88, *p* = 0.014) were both associated with lower odds of being foreign-born compared to full-time employment.

Geographically, compared to Kanto (Greater Tokyo), residence in Kansai (Osaka and Kyoto) (AOR = 0.67, 95% CI: 0.46–0.98, *p* = 0.039), Kyushu/Okinawa (AOR = 0.24, 95% CI: 0.11–0.52, *p* < 0.001), Tohoku (Northern Japan) (AOR = 0.13, 95% CI: 0.03–0.55, *p* = 0.005), and Hokkaido (AOR = 0.29, 95% CI: 0.09–0.93, *p* = 0.038) were associated with lower odds of being foreign-born.

**Table 2:** Multivariable logistic regression examining sociodemographic correlates of being foreign-born (n = 7662).

	Univariable Regression		Multivariable Regression	
Variable	OR (95% CI)	*p*-Value	AOR (95% CI)	*p*-Value
**Age (years)**				
Age	0.94 (0.93–0.95)	<0.001	0.94 (0.93–0.95)	<0.001
**Sexuality**				
Gay	REF		REF	
Bisexual	0.75 (0.53–1.06)	0.102	0.58 (0.40–0.85)	0.005
Something else	0.61 (0.28–1.30)	0.201	0.36 (0.14–0.91)	0.032
**Recruitment group**				
In-person recruitment	REF		REF	
App advertisement (male model)	1.27 (0.61–2.66)	0.520	1.63 (0.70–3.79)	0.256
App advertisement (community)	2.06 (1.01–4.21)	0.048	2.73 (1.20–6.18)	0.016
**Employment**				
Full-time employee	REF		REF	
Part-time/casual employee	0.38 (0.19–0.78)	0.008	0.38 (0.16–0.88)	0.025
Student	0.59 (0.31–1.12)	0.105	0.92 (0.47–1.77)	0.797
Unemployed	2.45 (1.66–3.62)	<0.001	0.93 (0.46–1.91)	0.854
Other	0.46 (0.29–0.72)	0.001	0.52 (0.31–0.88)	0.014
**Income**				
<2 million yen	REF		REF	
No income	1.01 (0.48–2.14)	0.970	0.93 (0.43–2.00)	0.853
2–5.99 million yen	0.79 (0.54–1.15)	0.217	0.96 (0.53–1.71)	0.878
6 million yen+	0.84 (0.55–1.29)	0.430	1.23 (0.65–2.33)	0.529
**Residential location**				
Kanto	REF		REF	
Kansai	0.67 (0.47–0.95)	0.027	0.67 (0.46–0.98)	0.039
Chubu	0.64 (0.44–0.93)	0.019	0.75 (0.50–1.12)	0.158
Kyushu/Okinawa	0.21 (0.10–0.45)	<0.001	0.24 (0.11–0.52)	<0.001
Tohoku	0.13 (0.03–0.52)	0.004	0.13 (0.03–0.55)	0.005
Chugoku	0.41 (0.17–1.00)	0.050	0.46 (0.18–1.13)	0.091
Hokkaido	0.25 (0.08–0.78)	0.018	0.29 (0.09–0.93)	0.038
Shikoku	0.16 (0.02–1.13)	0.066	0.18 (0.03–1.31)	0.090

### 4.3. Country of Birth and Health/HIV-Related Behaviours (Table 3)

Table 3 shows that compared with men born in Japan, foreign-born participants had higher odds of HIV testing in the past 6 months (AOR = 1.75, 95% CI: 1.33–2.32, *p* < 0.001), HIV testing in the past 12 months (AOR = 1.98, 95% CI: 1.50–2.61, *p* < 0.001), and ever having tested for HIV (AOR = 2.40, 95% CI: 1.66–3.46, *p* < 0.001). There were no significant differences between groups for UAI with a casual partner or suicidal ideation in the past two weeks.

Foreign-born men were more likely to have ever wanted psychiatric or psychosomatic treatment but given up (AOR = 1.52, 95% CI: 1.10–2.09, *p* = 0.011), but did not differ significantly from Japanese-born men in terms of ever receiving psychiatric/psychosomatic treatment.

**Table 3:** Multivariable logistic regression examining associations between country of birth and health and HIV risk-taking and protective behaviours *.

	Univariable Regression		Multivariable Regression	
Variable	OR (95% CI)	*p*-Value	AOR (95% CI)	*p*-Value
HIV test in past 6 months	1.98 (1.52–2.57)	<0.001	1.75 (1.33–2.32)	<0.001
HIV test in past 12 months	2.13 (1.65–2.75)	<0.001	1.98 (1.50–2.61)	<0.001
Ever tested for HIV	1.58 (1.17–2.14)	0.003	2.40 (1.66–3.46)	<0.001
UAI with casual partner (past 6 months)	0.85 (0.63–1.15)	0.282	0.91 (0.65–1.26)	0.557
Suicidal ideation (past two weeks)	1.05 (0.80–1.39)	0.706	0.94 (0.69–1.28)	0.685
Ever received psychiatric or psychosomatic treatment	0.74 (0.56–0.98)	0.033	0.84 (0.62–1.13)	0.246
Ever wanted psychiatric treatment but gave up	1.60 (1.19–2.16)	0.002	1.52 (1.10–2.09)	0.011

* Controlling for Age, Sexuality, Recruitment group, Employment, Income and residential location.

### 4.4. Country of Birth and Disclosure, Social Capital, and Community Engagement (Table 4)

Table 4 shows that foreign-born men had higher odds of having disclosed their sexuality to any friends (AOR = 1.96, 95% CI: 1.34–2.87, *p* = 0.001) and to family (AOR = 1.93, 95% CI: 1.46–2.55, *p* < 0.001). They were less likely to be aware of an LGBT/AIDS prevention organisation (AOR = 0.48, 95% CI: 0.36–0.65, *p* < 0.001), but more likely to have participated in a programme run by such an organisation in the past year (AOR = 2.35, 95% CI: 1.45–3.80, *p* < 0.001).

Regarding social capital, foreign-born men had higher odds of high gay social capital (AOR = 1.56, 95% CI: 1.08–2.25, *p* = 0.018) and high heterosexual social capital (AOR = 2.07, 95% CI: 1.37–3.13, *p* = 0.001) compared to Japanese-born MSM.

**Table 4:** Multivariable logistic regression examining associations between country of birth and disclosure, social capital, and community engagement outcomes *.

	Univariable Regression		Multivariable Regression	
Variable	OR (95% CI)	*p*-Value	AOR (95% CI)	*p*-Value
Disclosed sexuality to any friends	2.40 (1.68–3.44)	<0.001	1.96 (1.34–2.87)	0.001
Disclosed sexuality to any family	2.09 (1.62–2.71)	<0.001	1.93 (1.46–2.55)	<0.001
Aware of LGBT or AIDS prevention organisation	0.43 (0.33–0.57)	<0.001	0.48 (0.36–0.65)	<0.001
Participated in LGBT or AIDS prevention programme (past 12 months)	2.14 (1.39–3.30)	0.001	2.35 (1.45–3.80)	<0.001
Low gay social capital	REF		REF	
Medium gay social capital	1.03 (0.75–1.41)	0.863	0.95 (0.67–1.34)	0.764
High gay social capital	1.72 (1.24–2.39)	0.001	1.56 (1.08–2.25)	0.018
Low heterosexual social capital	REF		REF	
Medium heterosexual social capital	1.56 (1.08–2.25)	0.017	1.31 (0.88–1.93)	0.183
High heterosexual social capital	2.73 (1.87–3.98)	<0.001	2.07 (1.37–3.13)	0.001

* Controlling for Age, Sexuality, Recruitment group, Employment, Income and residential location.

## 5. Discussion

This study provides the first large-scale national examination of the sociodemographic, social, and health, wellbeing and HIV risk correlates of being foreign-born among MSM living in Japan. Rather than identifying elevated mental health disparities or poorer HIV prevention engagement, the findings reveal a pattern of differential social positioning shaped by age, region, employment, social capital, and access to institutional and community resources. In doing so, this research extends prior Japanese MSM research [12,13,25] by demonstrating how migration status intersects with both gay and heterosexual social capital to shape health access and wellbeing outcomes.

Foreign-born MSM were disproportionately younger and were concentrated in metropolitan regions, particularly Kanto (Greater Tokyo), with substantially lower odds of residing in southern (Kyushu and Okinawa) or northern (Tohoku and Hokkaido) Japan. This pattern likely reflects labour migration pathways and access to sexual minority infrastructure in large urban centres, consistent with research on sexual minority migration and settlement in Japan [18,34]. Employment status further highlighted structural differentiation. Foreign-born MSM were less likely to be in part-time or casual employment compared with full-time employment, while those classified as unemployed often showed elevated odds of being foreign-born, though only approached statistical significance. These findings suggest a pattern whereby foreign-born MSM are overrepresented both among securely employed full-time workers and among those experiencing labour market exclusion. Such polarisation is consistent with Japan’s migration system, which privileges skilled and professional migrants while simultaneously producing insecurity among those with restricted work rights or unstable visa conditions [35]. These employment dynamics likely intersect with access to health care, social services, and community participation [36].

Importantly, foreign-born MSM demonstrated higher engagement with HIV testing. This contrasts with common deficit narratives that position migrant MSM as under-tested or disengaged from prevention services. Instead, these findings suggest that foreign-born MSM who are able to access health systems may demonstrate strong preventive health behaviours, consistent with international research showing that migrant MSM often exhibit equal or higher testing uptake when structural barriers are mitigated [37]. In the Japanese context, this pattern may also reflect comparatively low levels of HIV testing among MSM overall, with Japan recording the lowest HIV testing prevalence in the Asia Pacific MSM Internet Survey [38]. These results reinforce the need to distinguish between access to services and willingness to engage with them.

The social capital analyses provide important insight into these dynamics. Drawing on a Bourdieusian framework, the findings indicate that while both heterosexual and gay social capital were associated with being foreign-born, heterosexual social capital was more strongly associated. This pattern aligns with previous findings in Japan, which demonstrated that heterosexual and gay social capital operate as distinct forms of capital with different health implications for MSM [13,39]. For foreign-born MSM, heterosexual social networks may function as key sources of economic stability, housing, and legal or bureaucratic navigation, particularly in a context where formal migrant support structures remain limited. However, reliance on heterosexual social capital may also constrain access to culturally specific sexual health information and community-based HIV prevention pathways.

This interpretation is further supported by findings related to community engagement. Foreign-born MSM were more likely to be recruited via community-oriented advertising than through in-person recruitment, suggesting that inclusive, non-sexualised messaging may be particularly effective for reaching this population. At the same time, awareness of LGBT or AIDS prevention organisations was lower among foreign-born MSM, even as participation in such programmes, once accessed, was strongly positively associated. This suggests that foreign-born MSM who successfully navigate entry points into community organisations may engage deeply, but face barriers to initial awareness and access. Similar patterns have been observed in international studies of migrant sexual minorities, where community engagement is often high among those who connect, but overall reach is constrained by language, information gaps, and weak bridging capital [37,39].

Disclosure patterns further complicate this picture. Foreign-born MSM were more likely to have disclosed their sexuality to friends and family, yet disclosure did not uniformly translate into greater access to institutional or community resources. This reinforces the importance of distinguishing between symbolic visibility and the possession of usable social capital. Prior work has shown that disclosure alone does not guarantee access to supportive networks, particularly where social ties lack the cultural or institutional capital necessary to facilitate health system navigation [36].

A key structural vulnerability identified in this study relates to mental health care access. Foreign-born MSM were significantly more likely to report having wanted psychiatric or psychosomatic treatment but having given up on accessing care. Notably, this pattern occurred in the absence of higher reported suicidal ideation, suggesting that disparities may arise not from elevated distress, but from barriers encountered during help-seeking. This finding is consistent with international evidence documenting unmet mental health needs among migrant populations, driven by language barriers, uncertainty around eligibility, cost concerns, and fear of discrimination in clinical settings [6,40]. In Japan, where mental health care remains highly medicalised and difficult to navigate without prior institutional knowledge, these barriers may be particularly salient for foreign-born MSM [36].

Taken together, these findings challenge assumptions that foreign-born MSM in Japan represent a higher-risk-taking or less engaged population. Instead, they point to a group that demonstrates strong preventive health behaviours, including higher HIV testing uptake, alongside limited access to community infrastructure and mental health services. These patterns underscore the importance of addressing structural and informational barriers, strengthening culturally and linguistically accessible services, and recognising the distinct roles played by gay and heterosexual social capital networks in shaping health outcomes. Improving pathways into community organisations and reducing attrition from mental health care will be essential for ensuring that prevention and wellbeing strategies are equitable and responsive to the diversity of MSM living in Japan. In a context of increasingly exclusionary and racialised public discourse surrounding foreign nationals in Japan, evidence-based approaches to health policy are essential to avoid reinforcing stigma and marginalisation.

### Limitations

Several limitations should be acknowledged when interpreting these findings. First, the cross-sectional design precludes causal inference, and all data were self-reported. The survey was administered in Japanese and, although some participants born abroad may have used translation tools or assistance, differences in comprehension cannot be ruled out. This language requirement possibly excludes foreign-born MSM with limited Japanese proficiency, who may face greater structural barriers, exclusion or discrimination, and reduced access to social and community resources. As a result, the experiences of the most marginalised foreign-born MSM may be underrepresented in this study. Future research should prioritise multilingual data collection strategies to better capture the diversity of migration-related experiences among MSM in Japan. In addition, the binary measure of country of birth does not capture citizenship, ethnicity, length of residence, or visa status, limiting the ability to examine heterogeneity within the foreign-born group. Importantly, some participants classified as foreign-born may have been Japanese nationals born overseas, further highlighting the diversity of experiences encompassed by this category.

Second, as data were collected primarily through geo-social networking applications, the sample may not fully represent all MSM in Japan. Individuals with limited internet access or lower engagement with online platforms, including some older MSM, may be underrepresented. Privacy concerns, particularly among those who have not disclosed their sexuality, may also have discouraged participation. However, given that many participants in this study had not disclosed their sexual sexuality to their families yet still chose to complete the survey, it appears that privacy concerns did not constitute a major barrier for a substantial proportion of respondents.

Third, the study relied on convenience sampling, which is commonly used in research involving hard-to-reach or stigmatised populations such as MSM and people who use substances [41,42,43]. Although this limits generalisability, similar levels of suicidal ideation and HIV testing have been reported among MSM in previous Japanese studies, supporting the broader relevance of the findings [13,19]. Despite these limitations, this study represents the largest survey to date of MSM born abroad in Japan, providing important insights into a population that remains underrepresented in empirical research.

Finally, reliance on self-reported behaviours and attitudes introduces the possibility of social desirability bias, particularly for sensitive topics [44]. Nevertheless, the anonymous online format of the survey is likely to have mitigated such bias compared with interviewer-administered or in-person data collection methods, thereby enhancing the credibility of participant responses.

## 6. Conclusions

These findings suggest that foreign-born MSM are neither uniformly marginalised nor uniformly privileged, but instead navigate complex configurations of advantage and constraint shaped by age, region of residence, employment, disclosure, and social networks. These findings highlight the need for structurally informed, community-based HIV prevention and mental health strategies that recognise the distinct social positioning and access patterns of foreign-born MSM in Japan. For health policy and prevention practice, these results underscore the importance of community-based approaches that recognise and build upon existing forms of social capital, while addressing structural barriers related to language, region of residence, and institutional access. In particular, sustained investment in gay and HIV prevention non-profit and community organisations is critical for reaching foreign-born MSM, including those who are culturally or linguistically diverse and less embedded in local networks. As Japan continues to grapple with persistently high proportions of AIDS diagnoses, understanding who foreign-born MSM are, and how they are positioned within social and structural hierarchies, is essential for developing equitable and effective health responses.

## Data Availability

Data is available to readers upon reasonable request due to privacy restrictions.
